# Research on the Intervention and Prevention of College Students' Mental Health Crisis From the Perspective of Ideological and Physical Education

**DOI:** 10.3389/fpubh.2022.905905

**Published:** 2022-06-22

**Authors:** Jiang Yu

**Affiliations:** College of Sports and Leisure, Guangdong Ocean University, Zhanjiang, China

**Keywords:** trust, wellbeing, mental wellbeing, preventive behavior, family support

## Abstract

Present study has aimed to understand the intervention and prevention of the mental health crisis of college students. For this purpose, this study has checked the effect of trust in wellbeing information on preventive behavior and mental wellbeing. The target population taken for this study is the students of colleges situated in Mainland in China. The data was collected from the 458 students of the college. Smart PLS has been employed on the data to get the results using partial least square structural equation modeling. For this purpose, the data were analyzed in two stages, i.e., measurement model stage and the structural model stage. Results of this study have revealed that trust in wellbeing information plays a significant and positive role in setting preventive behavior and the mental wellbeing of students. Further, it has also been revealed that preventive behavior also plays a significant and positive role in mental wellbeing. Additionally, preventive behavior has been found as an important mediating variable among the relationship of trust in wellbeing information and mental wellbeing. Moreover, family support is crucial by positively moderating the relationship between preventive behavior and mental wellbeing. Many practical implications have been found among which the foremost is that the education institutes must undertake those efforts that aim to ensure the fairness in the information spread regarding the mental wellbeing during seminars, workshops and administration should play a positive role responsible for strengthening the mental health of students by managing ideas, improving student education management, innovating management techniques and methods.

## Introduction

A great emphasis has been laid upon the comprehensive development of college students for cultivating high-quality qualified generation by promoting the reform of quality education and complying with the requirements of mental health development. Recently, mental health education has become a significant issue in college education (1). The college students have experienced distorted psychological events which lead to serious mental health issues among them. Improving mental health education in universities and colleges has been regarded as a fundamental aspect of education management (2). Colleges and universities have given great importance to mental health education. This education is conducive to maximizing the ideological and health factors among college students, enhancing the degree of cultural and spiritual aspects, creating a positive campus environment for students, and developing a positive learning environment (3). Additionally, the role of physical education is significant in improving the mental wellbeing of the students because physical education releases tension and mental fatigue.

Administrators of college students' education play a significant role in improving school management. The administrators are responsible for strengthening the mental health of students by changing managing ideas, improving student education management, innovating management techniques and methods, and promoting the development and progress of education management for college students (4). This can be done by providing physical education to students because this education is not only related to fitness and sport but also contributed toward the healthcare of students. The mental wellbeing of students is crucial, otherwise, students may engage in deviant or unpleasant activities in or outside the college. American College Health Association presented a report indicating that 14 percent of college students have experienced depression in the past 12 months while 17 percent of students are victims of anxiety, with trends rising (5). Moreover, 15 percent of college students' academic performance has been affected by depression, while, anxiety has affected ~23 percent of college students' academic performance. Therefore, the need to examine the strategies to promote mental wellbeing has aroused.

Additionally, mental health disorder has been seen to be positive among college students over the last 5 years (6). The practitioners have recognized that it is quite challenging to overcome common health problems. Generally, students have low mental health awareness and do not consider treatment for combating these issues but rather perceive these problems as normal college or academic stress, therefore, do not go for treatment (7). However, the students who consider treating their mental health issues often come across several barriers to access care, are skeptical about the treatment, and believe the mental health treatment to be inconvenient (8). It becomes evident that mental wellbeing is a significant feature for college students and analyzing this aspect is crucial for the students.

The issue of mental health has become significantly crucial due to the rise in suicidal cases, depression, and anxiety among the students (9). To combat these issues and improve mental wellbeing, students tend to seek information about mental wellbeing. They generally come across authentic or fake information that circulates over the internet (10). Authentic information is perceived as useful for the students, thus increasing trust in wellbeing information. However, the students who rely on fake information may not trust the wellbeing information. Moreover, mental wellbeing is a sensitive topic as serious consequences are attached to this phenomenon. Therefore, trust in wellbeing information is important for the students which may help in overcoming serious health-related issues (8).

Individuals having mental issues often avoid getting treatment compliance due to their self-stigma issues (11). This self-stigma prevents the individuals to get proper treatment and improve their mental wellbeing because of high psychological distress and low self-esteem (12). However, the individuals who are not reluctant and are willing to improve their mental wellbeing, seek wellbeing information from different sources. If such individuals have high trust in wellbeing information, they would certainly overcome mental health issues (13). Nonetheless, trust in this type of information is very critical due to the sensitivity of the situation and changing the mindset of the individuals who are reluctant toward mental health treatment (14).

Compared to biological and intentional processes, health behavior has been widely regarded as a behavior for studying the impacts of mental wellbeing. Generally, there are two types of health-related behavior, i.e., risky health behavior and preventive health behavior. Risky health behavior involves the susceptibility of an individual toward an illness or a disease (5). While, preventive health behavior involves the maintenance or improvement of health, either within or outside the medical case vicinity (2). For the present study, preventive health behavior has been studied in terms of improving the mental wellbeing of college students.

Preventive health behavior enables individuals to take precautions to avoid any type of disease or illness. In the case of college students, the students tend to not burden themselves with academic pressures, therefore they induce preventive health behavior (15). Furthermore, the students also talk to their peers and close friends to prevent and protect their mental health. According to Broholm-Jørgensen et al. spending time with your close one can improve the mental wellbeing of the individuals (16). Additionally, students feel quite relieved by engaging in preventive health behavior because they take precautions before the situation gets worse (10).

In the case of improving mental wellbeing, the role of family support is quite significant, especially for students. Receiving family support from parents, siblings, or relatives improves outcomes, such as satisfaction, relived, and calmness, thus improving the overall mental wellbeing (17). Not only this, family support strengthens the relationship between family members which is integral to successful acculturation (18). The students who have family support from any member of the family make college life less stressful as compared to students who have no family support (19). This indicates that family support is an important component in improving the overall mental health or mental wellbeing of the students. The involvement of parents significantly impacts the students' educational aspirations, thus, creating better mental wellbeing for the students (20). Building social relationships with individuals in the college community may be difficult for both extroverts and introverts, however, strong ties with family members is more effective in this case. Moreover, family especially parents, can better understand the mental ability of their child, thus this enables the students to discuss the matters with their family. Family support enhances the chances of better mental wellbeing for college students (21). To the best of the present author's knowledge, there is no empirical evidence for assessing the relationship of mental wellbeing in terms of trust in wellbeing information and mental wellbeing. Moreover, Zhou examined the factors that prevent infection behavior among people with mental illness (22). The authors suggested incorporating other measurement methods (e.g., preventive behavior) as a mediator to support the findings of the study. Therefore, the present study examined the mediating role of preventive behavior in the relationship between trust in information wellbeing and mental wellbeing. Furthermore, limited literature is available in terms of analyzing the moderating role of family support. Thus, to bridge the gap in the human health literature, there is an urgent need to explore this moderator. To do so, the current study opted to examine the moderating role of family support in the relationship between preventive behavior and mental wellbeing.

The current study developed the research objectives that have to be addressed to fill the gap in the existing body of literature and contribute to the literature. The objectives of the study include: to examine the impact of trust in wellbeing information on mental wellbeing, to determine the influence of trust in wellbeing information on preventive behavior, to analyze the impact of preventive behavior on mental wellbeing, to examine the mediating role of preventive behavior in the relationship between trust in information wellbeing and mental wellbeing, and to determine the effect of family support as a moderator in the relationship between preventive behavior and mental wellbeing among college students.

## Theoretical Framework and Hypotheses Development

**Figure 1:**
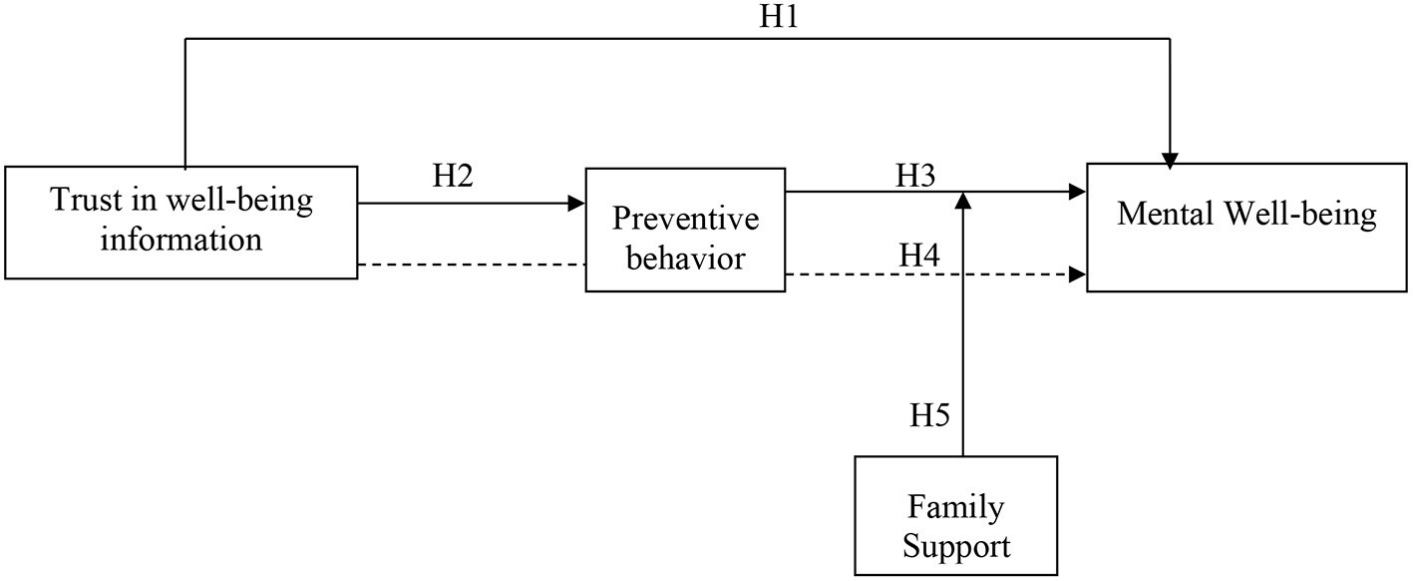
Theoretical framework.

The purpose of the current study is to research the intervention and prevention of college students' mental health crisis from the perspective of ideological and physical education. For this reason, the study has examined the impact of trust in wellbeing information on the mental wellbeing of college students in China. The study also analyzed the mediating role of preventive behavior in the relationship between trust in wellbeing information and the mental wellbeing of college students. Moreover, this study intended to determine the relationship between preventive behavior and the mental wellbeing of college studies. This framework of the study (see Figure 1) has been supported by two theories of human psychology and these theories are moral disengagement theory and wellbeing theory.

### Social Cognitive Theory

Albert Bandura developed the social cognitive theory in 1997. This theory explains that the influence of individual experiences, environmental factors, and the actions of others significantly impact individuals' health behavior (23). The social cognitive theory encompasses the importance of social support through self-efficacy, expectation, and utilizing observational learning to change an individual's behavior. The key indicators of social learning theory regarding individual behavior change incorporate self-efficacy, expectations, behavioral capacity, expectancies, observational learning, self-control, and reinforcements. Self-efficacy focuses on the belief of an individual that a behavior can be controlled and executed. Expectations highlight the results of a change of behavior (24). Behavioral capacity highlights the skills required to perform a behavior.

Considering the framework of the present study and the social cognitive theory, preventive health behavior has been taken as a mediator in the relationship between trust in wellbeing information and mental wellbeing. As explained earlier the health behavior of an individual is shaped by environmental factors. In the present study, the experience and environmental factors are the information that is generally acquired by the environment. Therefore, trust in information wellbeing is an environmental factor that has the potential to shape the behavior of the individual and exhibit preventive health behavior.

### WellBeing Theory

The wellbeing theory was developed by Seligman in 2011 (25). This theory proposes five key components of wellbeing are PEMRA i.e., positive emotion, engagement, relationships, meaning, and achievement. These indicators are independent of one another and they positively influence the wellbeing of an individual. The component of wellbeing that is related to the present study is relationships (26). This indicates points out the fact that feeling valued by others a having close bonds, increases the satisfaction level and improves the overall mental wellbeing. The innate need of an individual toward the relationships throughout their lives starts at a very early age. Moreover, one of the most fundamental needs of humans is close relationships. Studies demonstrated that relationship with close friends and family members increases the self-esteem of an individual, which eventually leads to better mental wellbeing (27). Additionally, college students with more friends and who have a close relationship with parents and siblings tend to be happier as opposed to students having no or weak relationships. Furthermore, good relationships among individuals increase the rate of happiness. In the light of wellbeing theory which posited that relationship with others is a fundamental aspect of wellbeing, therefore, the present study has incorporated family support as a moderator in the relationship between preventive behavior and mental wellbeing. As family support is crucial for improving wellbeing, this moderator tends to be suitable for the present study. Also, the theory elaborates that social relationship (family support) is essential for students' mental wellbeing.

### Trust in Wellbeing Information and Mental Wellbeing

The issue of mental health problems has been increasing over the years (28). The reasons for such issues are mainly related to external circumstances, internal crises, financial crises, the economy, etc. (29). For college students, mental health problems are subjected to academic pressures, a low level of stigma, uncertainty about a career path, etc. (30). These issues distort the mental health of the students and students become the victim of depression, anxiety, and behavioral problems. In such circumstances, individuals seek information from various sources including the internet, social media, psychiatrists, or even family or peers (31). Individuals having high trust in wellbeing information would generally have better mental wellbeing. Trust in any circumstance is significant for the individuals, otherwise, they indulge in repellent behavior (32). Wellbeing is one of the most commonly discussed topics in the present era, therefore, having high trust in information about such topics is essential for individuals.

Along with reliable information, fake information also circulates over the internet which hampers the trust of the individuals in the information (4). We acquire information 24/7 through social media platforms, television, and advertisements, therefore the issues of confirmation bias arise influencing the trust of the individual in information (33). The believability of fake information seems to become stronger and unavoidable with time (34). In the case of mental wellbeing, the information becomes more critical. Already the majority of the population is reluctant to get treatment for mental wellbeing (3). This becomes worst when such individuals come across fake information about wellbeing. Moreover, trust in wellbeing information can be developed if the individuals acquire information only from reliable and credible sources (29).

Fewer studies have been conducted on trust in wellbeing information and mental wellbeing. However, one related study was carried out by Lee who examined the role of institutional trust in the context of subjective wellbeing and mental health wellbeing (35). This empirical study demonstrated that institutional trust is critical for subjective wellbeing and mental health wellbeing. Moreover, Jain investigated the impact of trust in news on mental health and wellbeing (36). The study revealed that a high level of fake news led people to experience less satisfaction, happiness, and gratitude with more stress. The study indicates that fake news decreases the trust level in mental wellbeing information, ultimately influencing the mental wellbeing of the people. In a similar context, Defeyter et al. explained that trust is one of the most crucial factors in fostering the mental wellbeing of people (37). The trust can be on either information, people, institutes, or peers.

The management of the universities and colleges are seeking ways to improve the mental wellbeing of the students. In this regard, Khan et al. examined the impact of trust in information provided by the university and the psychological wellbeing of the students (38). The findings suggested that trust in information significantly impacts the psychological wellbeing of college students. This indicates that trust is a fundamental aspect of the mental wellbeing of the students. Critically, the trust factor is important when acquiring information on wellbeing because every person has a different perspective of wellbeing, therefore understanding wellbeing and implementing suitable ways to improve mental wellbeing comes with trust in wellbeing information (37). Limited research has been carried out in examining the relationship between trust in wellbeing information and the mental wellbeing of college students. Therefore, to bridge this gap in the literature, the presents study proposed the following hypothesis:

***H***_**1**_***:*** *Trust in wellbeing information plays a role in mental wellbeing*

### Trust in Wellbeing Information and Preventive Behavior

The mass media sources such as print, broadcast, and electronic media provide several types of information for the people. Particularly, health information-seeking behavior and having trust in such information are crucial for individuals (39). The increased accessibility and availability of information related to health and wellbeing have enabled people to focus on disease prevention. Consequently, people develop preventive behavior as a result of health information-seeking behavior (40). Individuals having high trust in wellbeing information, actively seek information to prevent themselves from any harmful diseases or illnesses. Moreover, trust in wellbeing information increases the level or degree of health consciousness among the individual. This type of information allows them to know about the diseases so that preventive action can be taken on time (41). A study by Park and Oh revealed that students rely more on online sources of wellbeing information because they can acquire abundant information from such sources (42). The reliable and accurate sources ultimately develop preventive health behavior among people (37). Individuals who have trust in wellbeing information can gain knowledge and awareness regarding wellbeing so that suitable preventive measures can be taken beforehand. In this regard, Plohl and Musil investigated the role of trust in science and information in the context of COVID-19 (43). The findings showed that trust in science and information influences compliance with COVID-19 prevention guidelines. The author indicated that trust consequently increases preventive behavior. In a similar context, another recent finding revealed by Zhao et al. showed that trust in media positively and significantly impacts infection mitigating behavior (44). The authors also discussed that higher risks of infection increase preventive mitigating behavior among people. Moreover, Lee asserted that preventive behavior is generally developed when the consequences of any disease are intolerable or uncontrollable (45). The researcher added that the people who engage in preventive behavior seek information about the disease, and act accordingly.

Unal et al. conducted an empirical analysis to explore the wellbeing of people residing in Turkey (46). The study aimed to investigate the precursors of preventive health behavior and revealed that preventive health behavior among people increases as a result of access to accurate information. Such information and preventive behavior together decrease the anxiety level of the people. Similarly, Broodryk and Robinson added that reliable information on wellbeing is a critical factor in fostering preventive health behavior, therefore, information must be acquired by reliable sources so that mental wellbeing can be improved (47). Limited literature is available with regards to the relationship between trust in wellbeing information and preventive behavior among college students. Therefore, the present study opted to investigate this relationship, hence the following hypothesis has been developed:

***H***_**2**_***:*** *Trust in wellbeing information plays a role in preventive behavior*

### Preventive Behavior and Mental Wellbeing

Psycho-social measures are always considered by human beings and such measures involve preventive behavior as very significant for diseases and illnesses (48). For instance, Butler et al. revealed how the level of awareness regarding respiratory infection and preventive behavior relates to the incidence of infection (17). Moreover, in the context of mental wellbeing, awareness and knowledge play a crucial role in developing preventive behavior among people (6). In particular, Kroke and Ruthig emphasized how psychological health functions as an essential factor in coping with mental illness (49). Preventive behavior is critical in improving mental wellbeing because this behavior enables individuals to take precautions to avoid any type of disease or illness. Moreover, Suhaimi asserted that prevention is better than cure because the person or individual takes action before the disease gets worse (10). Additionally, Shook et al. also argued that a disease can be avoided by people having preventive behavior because such individuals maintain or improve their health through different measures (50).

In the case of students, universities and colleges are striving to improve the mental wellbeing of the students. In this regard, Yildirim and Arslan suggested that one of the ways to nourish the mental wellbeing of college students is by developing preventive behavior among them (51). Furthermore, Rupert and Dorociak investigated the factors influenced by preventive behavior among college students (52). The study revealed that preventive behavior highly impacts the self-care and mental wellbeing of college students. However, preventive health behavior is not common among individuals, especially in terms of mental wellbeing. People avoid and are reluctant to go for mental wellbeing treatment because of their self-stigma (9). The college students perceived stress, anxiety, and depression as normal academic stress; therefore, they ignore their mental illness. As a result, they do not engage in preventive health behavior which further distorts their mental wellbeing (45).

Preventive health behavior significantly impacts the mental wellbeing of people, provided that they are aware of the issue (53). Moreover, Vann et al. claimed that fear of death after catching a disease also develops preventive health behavior among people (54). The people get worried about getting ill; therefore, they take precautionary measures to prevent the disease. Consequently, preventive health behavior improves the health of the individual. Very few studies have been conducted to investigate the direct relationship between preventive behavior and the mental wellbeing of college students. Hence, the present study aimed to fill this gap in the literature and proposed the following hypothesis:

***H***_**3**_***:*** *Preventive behavior plays a role in mental wellbeing*

### Mediating Role of Preventive Behavior

Preventive health behavior is developed as a result of health literacy, accessibility to information, and communication with others (37). Having low health literacy hampers the development of preventive behavior of individuals because of a lack of understanding, awareness, and knowledge related to health issues. According to different authors, health literacy comes with trust in information wellbeing and this subsequently improves the overall mental health of the people (50, 55). Park and Oh investigated the factors associated with preventive behavior among South Korean citizens (42). The study found that perceived severity and perceived susceptibility of the disease positively influence the preventive behavior of the citizens. Moreover, Vann et al. also examined the factors that are positively and significantly associated with preventive behavior toward mental wellbeing (54). The results showed that both perceived fear and accessibility of wellbeing information positively and significantly impact preventive behavior.

The mediating role of preventive health behavior has been explored in different contexts. For example, Suhaimi et al. analyzed the mediating role of health preventive behavior in the relationship between health literacy and wellbeing among adults in Hong Kong (10). The study suggested that health literacy is positively associated with wellbeing and health preventive behavior mediated this relationship. Similarly, Ayandele et al. also examined the mediating role of preventive health behavior in the relationship between fear of COVID-19 and mental wellbeing among Nigerians (56). This empirical investigation revealed that preventive health behavior is a significant mediator in the relationship between fear of COVID-19 and mental wellbeing among the people of Nigeria. Likewise, another recent study by Olapegba et al. aimed to investigate the relationship of fear of COVID-19 and psychological distress with the mediation of preventive health behavior (PHB) (57). The authors discussed that preventive health behavior significantly mediates the relationship between fear of COVID-19 and psychological distress.

Various studies have been conducted to examine the mediating role of preventive health behavior, however, most of these studies are carried out in the context of COVID-19 and other related diseases and infections. Very few studies explored preventive behavior as a mediator in the context of mental wellbeing. Therefore, the current study intended to investigate and analyze the mediating role of preventive behavior in the relationship between trust in wellbeing information and mental wellbeing. There is a dire need to investigate the mediating role of preventive behavior in the mental wellbeing context among college students, thus the following hypothesis has been developed:

***H***_**4**_***:*** *Preventive behavior mediates the relationship between trust in wellbeing information and mental wellbeing*

### Moderating Role of Family Support

Students face various challenges throughout their college life which resulted in a high level of anxiety, depression, and fear of failure. However, these challenges can be mitigated with the help of family support. To support this, Lukacs conducted a study to examine the relationship between family support and the mental health of students (58). The author discussed that family support is a major predictor of the mental wellbeing of students. Moreover, Khallad and Jabr analyzed the impact of perceived family support on college students' mental wellbeing (depression and stress) (59). The study found that the mental wellbeing of college students is improved through family support. A recent study carried out by Rahman et al. investigated the factors leading toward low mental wellbeing and suicidal behavior among university students of the UK (60). The empirical evidence showed that high academic pressure and lack of family support leads to low mental wellbeing and suicidal behavior.

Studies have been conducted to examine the moderating role of family support in the context of mental health. For example, Guo and Chen studied family support as a moderator in the relationship between psychological resources and the psychological wellbeing of individuals (61). The study found that family support is a strong moderator in this relationship. Another study that explored the moderating role of family support between stress and the mental wellbeing of students was conducted by Parka and Lee (62). Based on the results of the study, the authors explained that family support weakens the relationship between stress and mental wellbeing, such that family support improves wellbeing and reduces the level of stress among individuals. Furthermore, Nielsen et al. asserted that family support is a strong moderator between psychological capital and the subjective wellbeing of university students (63). House et al. added that support from instructors and family has been shown to impact and influence the subjective wellbeing of university students (21).

In the human psychology literature, empirical work has highlighted the moderating role of family support in fostering the impacts of academic stress on the subjective wellbeing of students. However, no prior study has analyzed how family support moderated the relationship between preventive behavior and the mental wellbeing of college students. This study intended to examine whether family support moderates the relationship between preventive behavior and mental wellbeing. This reasoning leads toward the development of the following hypothesis:

***H***_**5**_***:*** *Family support moderates the relationship of preventive behavior and mental wellbeing*

## Methodology

This study has incorporated the quantitative design for testing the hypotheses based on the primary data. This has been chosen because the variables have been quantified and checked their impact on each other according to the proposed framework (see Figure 1) supported by a review of literature conducted in section theoretical framework and hypotheses development. The present study checks the impact of trust in wellbeing information on mental wellbeing. It further checks the preventive behavior on mental wellbeing and how family support moderates this relationship. Therefore, the research philosophy followed in the present study is post-positivism. The research approach followed is deductive since it narrows down the broader concepts present in the literature (64). The population targeted in this study is the students enrolled in the colleges in Mainland in China. This particular sector has been chosen because they are growing in their careers and the importance of their wellbeing is unquestioned. The sampling technique chosen is non-probability sampling which is convenience sampling (65). They are easy to approach and the theme of the study was related to them so they took a keen interest in filling out the questionnaires. The sample taken was 550 and the questionnaires had been distributed among the respondents. The questionnaires that produced usable responses were 458 (response rate = 83.2%).

Ethics has been taken into account while conducting this research. The potential respondents had been contacted in advance to coordinate with their corresponding program coordinators to check the availability of the respondents. After prior approval, the students were provided with the questionnaires and they have read the cover letter explaining the purpose of the study and how to fill it. The questionnaires were administered by the researcher themselves to avoid any ambiguity and misunderstanding while filling the questionnaires. The data was collected at one point in time thus following the cross-sectional empiricism. Details for the questionnaire have been given in the next section. The responses collected through data collection have been analyzed through the Smart PLS software for using the technique of partial least square structural equation modeling. This technique provides the researcher the independence to analyze the hypotheses by simultaneously running regression among all variables, small sizes, and no boundary for when and how the data was collected (66). This gives the results on the basis of covariance among the variables where normal distribution is not a condition (67).

### Instrument Development

The data was collected through the questionnaire. It was designed on a five point Likert scale where 1 is considered strong disagreement and 5 is considered as strong agreement. The details of the questionnaire are as follows. The variable trust in wellbeing information consisted of 4 items. The scale for trust in wellbeing information has been adapted from Soveri et al. (68). The dependent variable of the mental wellbeing consisted of five items that have been adapted from Darvishmotevali et al. (69). The mediating variable of preventive behavior consisted of four items that have been adapted from Claassen et al. (70). The moderating variable of family support consisted of four items that have been adapted from Qurban et al. (71). The internal consistency and validity of the scales have been checked in the following sections.

### Demographic Profile

First of all, the data obtained regarding the demographic profile has been analyzed with the help of frequencies and percentages. The first variable of gender was categorized into two i.e., males and females. Male and female involvement has been found to be equal. The second variable of education was categorized into high school and bachelor. Fifty nine percent of participants belonged to high school while the rest of 40% belonged to bachelors. The last variable of demography was based on the scores obtained by the students. It was categorized into four i.e., distinction, good, average, and below average. The highest participation was found by good and average achievers. The results for demography are given in Table 1.

**Table 1:** Demographics analysis.

**Demographics**	**Frequency**	**Percentage %**
**Gender**
Male	232	50.65
Female	226	49.34
**Education**
High school	271	59.17
Bachelors	187	40.82
**Average grades**
Distinction (above 90%)	78	17.03
Good (80–90%)	145	31.65
Average (60–80%)	139	30.34
Below Average (Below 60%)	96	0.96

## Data Analysis and Results

**Figure 2:**
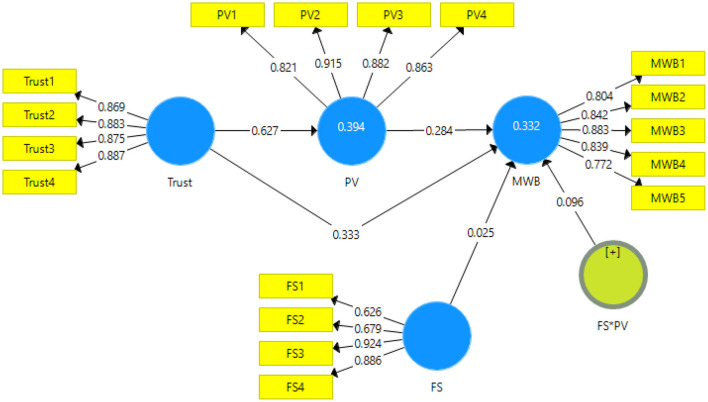
Output of measurement model. Trust, trust in wellbeing information; PV, Preventive behavior; MWB, Mental wellbeing; FS, Family Support; FS*PV, Moderating effect of family support.

**Figure 3:**
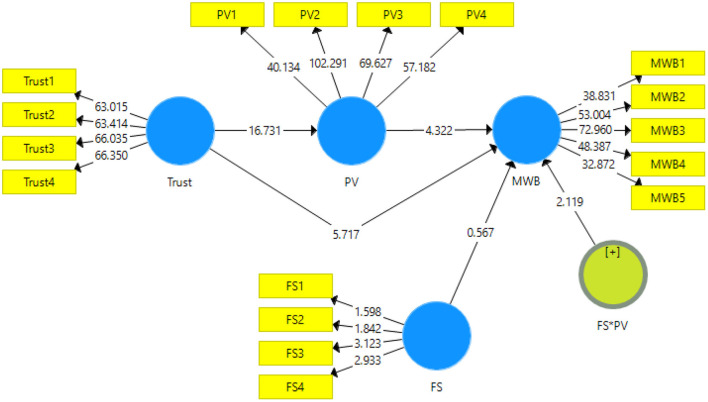
Output of structural model. Trust, Trust in wellbeing information; PV, Preventive behavior; MWB, Mental wellbeing; FS, Family Support; FS*PV, Moderating effect of family support.

The data has been analyzed using the partial least square structural equation modeling. It is usually analyzed in two stages. The first stage is model measurement (Figure 2) and the second stage is structural model measurement (Figure 3). The model measurement is done through the initial screening for validity and reliability of the scales. The reliability is checked through Cronbach alpha and composite reliability. The validity is checked through convergent (factor loadings, average variance extracted, variance inflation factor) and discriminant validity (heterotrait-monotrait ratio and Fornell and Larcker criteria) (72).

### Measurement Model

The output for the measurement model is given in Figure 2.

Table 2 shows the results for the internal consistency and reliability of the scales. The first criterion used is factor loading of the items for each variable. The acceptance criteria of the factor loadings are indicated as 0.6 in the literature. In this study, all the values obtained for factor loadings for each variable are above this criterion, hence, meeting the criteria and showing significant factor loading. The minimum value obtained in this study is 0.623 which is for FS1 item for the variable family support. This value though is also well above the cut off value. The acceptance criteria for Cronbach alpha and composite reliability is mentioned as being above 0.7. In this study, the minimum value for Cronbach alpha is 0.793 and for composite reliability is 0.826 thus indicating the scales used in this study are reliable. Furthermore, the average variance extracted, for the scales should be above 0.5 thus indicating the variance is explained more than the error (73).

**Table 2:** Convergent validity and reliability.

**Variables**	**Factor loadings**		**Cronbach alpha**	**Composite reliability**	**AVE**
Family support	FS1	0.623			
	FS2	0.679	0.881	0.865	0.623
	FS3	0.824			
	FS4	0.826			
Mental Wellbeing	MWB1	0.804			
	MWB2	0.842	0.888	0.916	0.687
	MWB3	0.883			
	MWB4	0.839			
	MWB5	0.772			
Preventive behavior	PV1	0.821			
	PV2	0.815	0.793	0.826	0.659
	PV3	0.882			
	PV4	0.863			
Trust in wellbeing	Trust1	0.869			
Information	Trust2	0.883	0.901	0.931	0.772
	Trust3	0.875			
	Trust4	0.887			

In this study, the discriminant validity has been checked through the most mainstream tests i.e., HTMT ratio and the Fornell and Larcker criteria. The HTMT ratio showed the discriminant validity as the values obtained for the test are below 0.85 (74). Results for HTMT ratio are given in Table 3. The highest value in this study is 0.685 for HTMT ratio which is for preventive behavior and trust in wellbeing information.

**Table 3:** Discriminant validity (HTMT ratio).

	**FS**	**MWB**	**PV**	**Trust**
**FS**
MWB	0.056			
PV	0.084	0.523		
Trust	0.083	0.553	0.699	

*Trust, Trust in wellbeing information; PV, Preventive behavior; MWB, Mental wellbeing; FS, Family Support*.

The Fornell and Larcker criteria were proposed by the researchers in order to obtain the discriminant validity of the variables (75). The acceptance criteria for the Fornell and Larcker criteria are it should show the highest values of each column at the top. The Fornell and Larcker criteria table is said to be confirming the discriminant validity when each column shows its highest number at the top (75). In this study, columns of all variables show the highest number of their column at the top (see Table 4). In Table 4 we can see that the highest value of each column is at the top. Like for the mental wellbeing column, he highest value is 0.829 which is at the top thus indicating the discriminant validity of the scale.

**Table 4:** Discriminant validity (Fornell and Larcker Criteria).

	**FS**	**MWB**	**PV**	**Trust**
FS	0.789			
MWB	0.050	0.829		
PV	0.036	0.494	0.871	
Trust	−0.019	0.525	0.627	0.878

*Trust, Trust in wellbeing information; PV, Preventive behavior; MWB, Mental wellbeing; FS, Family Support*.

### Structural Model

The researchers in this study have used structural model to check the hypotheses if supported or not (66). The structural model is run using bootstrapping method of resampling at 95% confidence interval. The hypotheses are confirmed for their support or denial using t-stats and *p*-values. The output for the structural model is given in Figure 3.

This study has shown interesting results for the hypotheses. The first hypothesis about the effect of trust in wellbeing information on mental wellbeing has been accepted (*t* = 5.717 and *p* = 0). The second hypothesis of the study about the effect of trust in wellbeing information on preventive behavior has been accepted (*t* = 16.731 and *p* = 0). The third direct effect was about the impact of preventive behavior on mental wellbeing has also been accepted with significant results (*t* = 4.322 and *p* = 0). Results for the direct effects can be seen in Table 5.

**Table 5:** Direct effects.

**Paths**	**H**	**β**	**SD**	**T-statistics**	***P***
Trust -> MWB	H_1_	0.332	0.058	5.717	0.000
Trust -> PV	H_2_	0.630	0.038	16.731	0.000
PV -> MWB	H_3_	0.284	0.066	4.322	0.000

*Trust, Trust in wellbeing information; PV, Preventive behavior; MWB, Mental wellbeing; FS, Family Support*.

Table 6 shows the indirect effects of the study. The first indirect effect is for the mediating role of preventive behavior between the relationship of trust in wellbeing information and mental wellbeing. This has been accepted showing significant results (*t* = 4.109 and *p* = 0). The second is about the moderating effect of family support on the relationship of preventive behavior on mental wellbeing. This hypothesis is also accepted showing significant augmenting effects on this relationship (*t* = 2.119 and *p* = 0.035). As all the values for *p* are <0.05, therefore, hypotheses are supported by the data.

**Table 6:** Indirect effects.

**Paths**	**H**	**β**	**SD**	**T-statistics**	***P***
Trust -> PV -> MWB	H_4_	0.179	0.043	4.109	0.000
FS*PV -> MWB	H_5_	0.103	0.045	2.119	0.035

Trust, Trust in wellbeing information; PV, Preventive behavior; MWB, Mental wellbeing; FS, Family Support; FS*PV, Moderating effect of family support.

## Discussion

The gap in the human psychology literature related to mental wellbeing has been bridged by collecting data from college students studying in China. The present study researched the intervention and prevention of college students' mental health crisis from the perspective of ideological and physical education. Initially, some direct relationships were studied, for example, the impact of trust in wellbeing information on mental wellbeing, the impact of trust in wellbeing information on preventive behavior, and the influence of preventive behavior on the mental wellbeing of college students studying in China. The study also determined the mediating role of preventive behavior in the relationship between trust in wellbeing information and the mental wellbeing of college students. The moderating role of family support was also studied in the relationship between preventive behavior and the mental wellbeing of college students. The result of the study provided great insights and recommendations for colleges.

The first hypothesis (H1) of the study posited that trust in wellbeing information plays a role in the mental wellbeing of college students. This hypothesis was accepted. These results are harmonious with the findings of Jain who investigated the impact of trust in news on mental health and wellbeing (36). The study revealed that a high level of fake news led people to experience less satisfaction, happiness, and gratitude with more stress. The reason is the factor of trust in information is crucial in mental wellbeing because this matter is sensitive and already the majority of the population show reluctance toward the treatment of mental wellbeing.

The second hypothesis (H2) of the study was accepted which posited that trust in wellbeing information plays a role in the preventive behavior of college students. The findings are in synchrony with the results obtained by Plohl and Musil which showed that trust in science and information influences compliance with COVID-19 prevention guidelines (43). The possible reason is that preventive behavior develops as a result of trust in information because people search for information to gain knowledge and understating about mental wellbeing. The third hypothesis (H3) of the study was also accepted which posited that preventive behavior plays a role in the mental wellbeing of college students. Similar results were found by Unal et al. who investigated the precursors of preventive health behavior and revealed that preventive health behavior among people increases as a result of access to accurate information (46). This is because preventive behavior focuses on improving and maintaining mental wellbeing, therefore preventive behavior and mental wellbeing have a significant and positive relationship. The fourth hypothesis (H4) of the study was also accepted which postulated that preventive behavior mediates the relationship between trust in wellbeing information and mental wellbeing of college students. These results are harmonious with the findings of Ayandele et al. who found that preventive health behavior is a significant mediator in the relationship between fear of COVID-19 and mental wellbeing among the people of Nigeria (56). Trust in wellbeing information increases preventive behavior because people understand the concept of mental wellbeing, this, in turn, improves the mental wellbeing of people. The fifth hypothesis (H5) of the study was also accepted which postulated that family support mediates the relationship between preventive behavior and mental wellbeing of college students. The findings are in synchrony with the results obtained by Nielsen et al. who asserted that family support is a strong moderator between psychological capital and subjective wellbeing of university students (63). The reason is that relationship and strong bonds with family members positively influences and improves the mental wellbeing of individuals. In the case of college students, the students can share their problems with family members which results in improved mental wellbeing.

### Practical Implications

This study offers many implications for the college administration and the educational policymakers. The administrators are responsible for strengthening the mental health of students by changing managing ideas, improving student education management, innovating management techniques and methods, and promoting the development and progress of education management for college students. First of all, it is obligatory for the educational institutes to undertake those efforts that aim to ensure fairness in the information spread regarding mental wellbeing. This is imperative that colleges hold different workshops and seminars regarding student counseling and other challenges; it is important that mental wellbeing is also touched as it is basic to human emotional and mental health. Since the study has shown that trust of people in the information spread has a direct positive and boosting relationship with the mental wellbeing henceforth, in colleges the responsibility lies with the management to make sure the information spread is authentic and reliable so people can easily trust it. Secondly, it is also useful for the organizations that they can prevent the behavior of the students other than negative reinforcement, if they are counseled thoroughly and continuously, it would help them detain themselves from engaging in hazardous behaviors like smoking, drugging, and other immoral behaviors. Thirdly, the responsibility lies with the families of the students as well that their support is crucial for their children in achieving wholesome mental wellbeing and emotionally stable personalities. Family support enhances the effect of preventive behavior on mental wellbeing showing how adolescents groom if they are taking adequate support from their families.

## Theoretical Contribution

The present study has made certain theoretical contributions as well to the body of literature in organizational psychology and education. First of all, the trust in wellbeing information has been found to have a positive and significant role in attaining wholesome mental wellbeing among college students. Secondly, it has also been found that trust in wellbeing information plays a positive and significant role in preventive behavior. Thirdly this prevents behavior also contributes significantly to attaining mental wellbeing. Furthermore, preventive behavior is found to be an important mediating variable for the relationship of trust in wellbeing information and mental wellbeing. Moreover, family support has been found to augment the role of preventive behavior in achieving mental wellbeing.

## Limitations and Suggestions for Future Studies

Along with practical and theoretical contributions, the study embeds a few limitations as well. First of all, the study focuses on the education sector only which limits its scope. However, mental wellbeing is a basic right of every human being. Therefore, it is important to spread the scope of the study by replicating this study in other corporate industrial sectors as well especially the lower management of the organizations. Because they are relatively more deprived of their rights in the organizations and are underpaid. Therefore, it will give us strong arguments regarding the importance of trust in wellbeing information and its role in achieving mental wellbeing. Secondly, the present study is limited to the Mainland in China. it is recommended to conduct this study in Europe and America as well, so the results could be compared and better conclusions could be given based on extensive findings. Furthermore, it will also make the study more comprehensive when data from different countries at different points of time will be taken giving more insights into the living patterns in different continents.

## Conclusion

Recently colleges and universities are giving great importance to the mental health education and overall wellbeing of the students. This education is conducive to maximizing the ideological and health factors among college students, enhancing the degree of cultural and spiritual aspects, creating a positive campus environment for students, and developing a positive learning environment. Therefore, the present study has formulated the framework based on the trust in wellbeing information and the mental wellbeing of students. The results of the study have shown interesting results like the significant role of trust in wellbeing information and preventive behavior in the mental wellbeing of the students. The study has also shown that trust in wellbeing information plays a significant role in preventing behavior among the students. Furthermore, it has also been found that preventing behavior is an important mediating factor between trust in wellbeing information and mental wellbeing. Alongside, another important revealing result of the study is the critical moderating role of family support to augment the effect of preventive behavior in mental wellbeing. The study has also shown many implications for the management and administration of the teaching institutes that can immensely contribute to obtaining their student's mental wellbeing.

## Data Availability Statement

The original contributions presented in the study are included in the article/supplementary material, further inquiries can be directed to the corresponding author.

## Ethics Statement

The studies involving human participants were reviewed and approved by Guangdong Ocean University, China. The patients/participants provided their written informed consent to participate in this study. The study was conducted in accordance with the Declaration of Helsinki.

## Author Contributions

JY conceived, designed the concept, and wrote the paper. The author read and agreed to the published version of the manuscript.

## Funding

This study was funded by 2021 Cooperative Education Project of Industry and University of Higher Education Department of Ministry of Education (202102283093) and 2020 Education and Teaching Reform Project of Guangdong Ocean University (580320134).

## Conflict of Interest

The author declares that the research was conducted in the absence of any commercial or financial relationships that could be construed as a potential conflict of interest.

## Publisher's Note

All claims expressed in this article are solely those of the authors and do not necessarily represent those of their affiliated organizations, or those of the publisher, the editors and the reviewers. Any product that may be evaluated in this article, or claim that may be made by its manufacturer, is not guaranteed or endorsed by the publisher.
